# Measured, modeled, and causal conceptions of fitness

**DOI:** 10.3389/fgene.2012.00196

**Published:** 2012-10-23

**Authors:** Marshall Abrams

**Affiliations:** Department of Philosophy, University of Alabama at BirminghamBirmingham, AL, USA

**Keywords:** fitness, natural, selection, probability, darwinian evolution, *F*_*ST*_, simulations, causality

## Abstract

This paper proposes partial answers to the following questions: in what senses can fitness differences plausibly be considered causes of evolution?What relationships are there between fitness concepts used in empirical research, modeling, and abstract theoretical proposals? How does the relevance of different fitness concepts depend on research questions and methodological constraints? The paper develops a novel taxonomy of fitness concepts, beginning with *type fitness* (a property of a genotype or phenotype), *token fitness* (a property of a particular individual), and *purely mathematical fitness*. Type fitness includes *statistical type fitness*, which can be measured from population data, and *parametric type fitness*, which is an underlying property estimated by statistical type fitnesses. Token fitness includes *measurable token fitness*, which can be measured on an individual, and *tendential token fitness*, which is assumed to be an underlying property of the individual in its environmental circumstances. Some of the paper's conclusions can be outlined as follows: claims that fitness differences do not cause evolution are reasonable when fitness is treated as statistical type fitness, measurable token fitness, or purely mathematical fitness. Some of the ways in which statistical methods are used in population genetics suggest that what natural selection involves are differences in parametric type fitnesses. Further, it's reasonable to think that differences in parametric type fitness can cause evolution. Tendential token fitnesses, however, are not themselves sufficient for natural selection. Though parametric type fitnesses are typically not directly measurable, they can be modeled with purely mathematical fitnesses and estimated by statistical type fitnesses, which in turn are defined in terms of measurable token fitnesses. The paper clarifies the ways in which fitnesses depend on pragmatic choices made by researchers.

## 1. Introduction

Evolutionary and population genetics use a wide variety of seemingly incompatible concepts of fitness. Stearns famously defined fitness as “Something everyone understands but no one can define precisely” (Stearns, 1976, p. 4). The need for systematic discussion of relationships between fitness definitions and their roles in research contexts has not been exhausted by past work, however. My purpose here is to make a narrow contribution toward the goal of understanding fitness. My strategy will use a novel classification of fitness concepts. Distinctions similar to some of mine exist in the literature, but the full taxonomy has not, to my knowledge, been previously applied. While I don't claim that my classification will cover every significant fitness concept in use today, I believe that it imposes a useful order on most practical fitness concepts, and that it helps advance our understanding of the roles that fitness concepts play.

**Figure 1:**
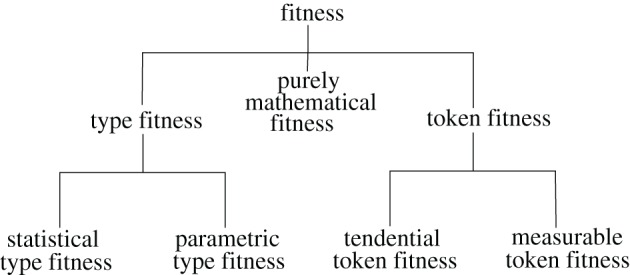
**Classes of fitness concepts defined in the text**. Lines connect classes to their subclasses.

I'll use my classification to argue for ways of specifying (a) senses of fitness that imply that it depends on pragmatic aspects of scientific practice and (b) senses of fitness which might allow fitnesses to be viewed as perfectly objective characteristics of elements of biological populations. Specifically, I'll define a contrast between *token fitness* and *type fitness*, subdividing the former category into *measurable token fitness* and *tendential token fitness*, and the latter into *statistical type fitness* and *parametric type fitness* (Figure 1). What I'll call *purely mathematical fitnesses* are often used ambiguously, indeterminately playing a role in definitions of different kinds of fitness. Since some readers may want to have a better idea of the purpose to which this relatively complex classification system is to be put, I'll summarize the main claims to be made in the paper before defining the terms.

Stearns also remarked that “Some disagreement over fitness measures can be avoided by asking ‘What questions do you want to answer and with which assumptions are you satisfied?’” (Stearns, 1992, p. 33). Different fitness measures clearly seem relevant in different research contexts, but Stearns' remark might be taken as suggesting that purely subjective choice infects scientific objectivity. A more precise characterization of the situation comes from suggesting that an appropriate fitness measure depends, first, on a *research question*—a conception of what properties of what population it is that we want to investigate—and on a set of *methodological constraints*—available data, analytical methods, or computational resources, and a desired degree of confidence in the results (cf. Stearns, 1976, 1992; de Jong, 1994; Ariew and Lewontin, 2004; Abrams, 2009b). Methodological constraints, especially, might be thought to introduce a subjective element into investigations of evolutionary processes. However, it's reasonable to think that the processes which are characterized by means of fitness measures can be perfectly objective. I believe that the distinctions made below between different types of fitness concepts can help sort out the apparent conflict between these intuitions.

Natural selection surely has to do with differences in fitnesses, and is reasonably thought to be a kind of cause of evolution, as I'll explain below. Yet some authors have argued that fitness differences do not cause evolution. Others have defined fitness in such a way that it is hard to understand how fitness is connected to natural selection. I'll argue that the senses in which fitness and natural selection cause evolution can be clarified using the fitness categories defined below. Moreover, understanding the senses in which fitness is or is not plausibly causal in nature will help us clarify relationships between research questions, methodological constraints, and fitness concepts.

Some of the paper's conclusions can be summarized as follows: when fitness is defined in terms of measurement, or when it is defined in a purely mathematical sense, then fitness differences do not cause evolution. It is only when fitness is defined as an underlying characteristic of something—an individual, a population, or a type—that fitness might be the sort of property which could conceivably cause evolution. Such underlying characteristics usually cannot be directly measured, although they can be estimated using measured properties. More specifically, using the terms mentioned above and defined below, I'll argue for the following points:

1. Claims that fitness differences do not cause evolution may be reasonable when fitness is treated as statistical type fitness (which is defined in terms of measurement) or as purely mathematical fitness.
2. Natural selection has to do, fundamentally, with differences in type fitness, so token fitnesses have only a derivative role in understanding natural selection
3. Some methods used in population genetics seem to require that what natural selection involves are differences in parametric type fitness (an underlying property).
4. It's reasonable to think that differences in parametric type fitness can cause evolution. I'll suggest a way that this claim might be understood, in terms of probabilities characteristic of particular heritable types in particular populations and environments.
5. Although parametric type fitnesses are typically not directly measurable, they can be modeled with purely mathematical fitnesses and estimated by statistical type fitnesses, which in turn are defined in terms of measurable token fitnesses. It may be reasonable to view parametric type fitnesses as defined by probability-weighted combinations of possible tendential token fitnesses.
6. Both research questions and methodological constraints affect which statistical type, measurable token, and purely mathematical fitnesses are relevant.
7. Research questions can select what kind of parametric fitness is of interest, but parametric fitnesses are independent of methodological constraints.

Obviously, these assertions require much unpacking and elaboration, and I don't claim that my arguments below are conclusive. However, I believe that earlier discussions of fitness have been limited by ignoring or conflating some fitness concepts.

I should note that my discussion is inspired by numerous uses of fitness concepts in both empirical and theoretical studies. However, since the points I make are quite general, applying to many different uses of fitness concepts at once, it will suffice to illustrate uses of such concepts using a small number of arbitrarily chosen citations. More examples could be given, but would be redundant and tedious.

Section 2 defines a hierarchical classification of fitness concepts. Section 3 describes the role of fitness in natural selection, and explains why it's reasonable to view natural selection as a cause of evolution. In section 4, I apply these ideas, arguing that fitness concepts in the classes I've defined play different roles in our understanding of natural selection in empirical populations. Section 5 then discusses the relationship between research questions, methodological constraints, and different kinds of fitness concepts, and section 6 summarizes the points made in the paper.

## 2. Classes of fitness concepts

In this section I'll define several classes of fitness concepts. I'll use these in the rest of the paper to help sort out some of the conflicting claims that have been made about fitness. Although there may be some fitness concepts which cannot be fit into this classification, I believe that the range of concepts to which it does apply make it a useful tool.

The distinctions which I'll make between different concepts labeled by “fitness” apply equally well to those labeled by many closely related terms, some with overlapping applications, including “selection coefficient,” “lifetime reproductive success,” “reproductive value,” “net reproductive rate,” “intrinsic growth rate,” “density-independent growth rate,” “Malthusian parameter,” “viability,” “number of offspring,” “selection differential,” “selection gradient,” “invasion fitness,” and others (e.g., Stearns, 1976, 1992; Lenski et al., 1991; de Jong, 1994; Michod, 1999; de Valpine, 2000; Cooper et al., 2003; Elena and Lenski, 2003; Ewens, 2004; Gillespie, 2004; Rice, 2004; Metz, 2008; Shaw et al., 2008). For the purposes of this essay, it will do no harm to refer to all such concepts, which in some way quantify differences in actual or probable contribution to the composition of a population at a later time, with the same term: “fitness.”

Some fitness concepts make no reference to anything directly involving survival or reproduction; if the point of such a fitness concept is to indicate likely changes in frequencies in a population, I consider it part of my focus. For example, Morris et al. (2012) define the fitness benefit of a loss-of-function mutation in terms of the resulting reduction in energy or resource use, if functions performed by the unmutated gene are also provided by other genes. Morris et al.'s discussion makes it clear that reduction of energy or resource use counts as a kind of fitness because of its likely effects on future frequencies. On the other hand, some fitness concepts are intended to capture the intuitive idea of “fit” with an environment, but that aspect of these concepts is not part of my focus. Thus I won't discuss the notion of “an adaptation” which is the primary focus of Reeve and Sherman (1993). However, I do include under my term “fitness” those concepts of “adaptedness” (Brandon, 1978, 1990; Byerly and Michod, 1991) which primarily concern future representation in a population. (Byerly and Michod also intend this concept to capture intuitions about fit with the environment.)

To avoid unnecessary complications, I focus here on the usual role of individuals in evolution by natural selection, without implying, for example, that notions of group selection or ideas about competition between cells within a multicellular organism are necessarily without significance. Throughout this essay I use “organism” to refer to an individual rather than to general characteristics of some class of individuals.

### 2.1. Type and token fitness

First, we can distinguish between what I'll call *type* and *token* fitness concepts. *Type fitness concepts* are ones which make fitness a property of organisms' properties or types—genotypes, phenotypes, etc. For example, population genetic models primarily use type fitness concepts. Thus in a single-locus haploid model, alleles represented in the model function as organisms' types. In diploid models, genotypes function as organisms' types, though with more complicated inheritance processes. If individuals are represented at all in population genetic models, it is typically only as bearers of the types which are the focus of the model. In some cases fitness may be attributed to genotypes involving many loci, or even to entire genomes (see below). Note that the fact that fitnesses are attributed to types does not imply that the environment plays no role in determining such fitness values. Nearly any fitness concept will make fitness values depend on the environment.

*Token fitness concepts* are those which make fitness a property of particular individual organisms. By this I mean that token fitnesses reflect an individual's complete set of genes, heritable and non-heritable phenotypic properties, and any details of surrounding environmental variations that can affect eventual reproductive success or success of descendants. These environmental circumstances may be different for every individual, even in an environment that would normally be treated as uniform. I elaborate on this point below.

[“Token” is a term for entities which are instances of a given type (e.g., Abrams, 2007). Token fitness concepts might also be called “individual fitness” concepts, since such fitnesses are properties of particular individuals. I avoid the term “individual fitness,” which is also used for certain type fitness concepts. For example, Michod writes “… fitness is often defined as the expected reproductive success of a type …. I refer to this notion of fitness as individual fitness” (Michod, 1999, p. 9).]

### 2.2. Measurable and tendential token fitness

Second, we can distinguish between two classes of token fitness concepts, which I'll refer to as *measurable* and *tendential* token fitness concepts. Note that token fitness concepts are often used in empirical studies, as de Jong (1994) notes. For example, Byars et al. (2010) estimated selection gradients for certain traits in a particular human population, in part using measurements of lifetime reproductive successes (LRS) of individual women in the population. LRS is a variety of measurable token fitness in this case. A measured LRS value is specific to an individual; it reflects the entire set of heritable and developmentally-determined traits, individual history, and circumstances that a particular individual in fact embodies or experiences. Measurable token fitnesses can even differ for developmentally identical clones if, for example, they experience different circumstances within the same overall environment after some point in time.

On the other hand, some authors use token fitness concepts which treat fitness as a general causal fact about a particular individual in its particular circumstances. Fitness in this sense may reflect, for example, the reproductive success that the individual is guaranteed to produce, or has a tendency to produce, or will probably produce. We can call such fitnesses *tendential* token fitnesses to distinguish them from measurable token fitnesses. Tendential token fitness concepts are intended to summarize all causal facts relevant to a particular individual's eventual evolutionary contribution, while measurable token fitness concepts capture an individual's actual evolutionary contribution. The distinction between these sorts of concepts is especially clear when a tendential fitness concept is intended to capture fundamental probabilistic causal facts which may or may not lead to particular outcomes. I suspect that tendential token fitness concepts are more common in informal statements by evolutionary biologists than in published work. Philosophers of biology have been more explicit in advocating the use of tendential token fitness concepts, as noted below.

### 2.3. Statistical type fitness

Third, it will be useful to distinguish between different classes of type fitness concepts, especially between what I'll call *statistical* and *parametric* type fitnesses. Here I use “statistical” and “parametric” in an extended sense derived from their usual use in statistics. A *statistical* type fitness concept is one that is defined in terms of one or more statistics which could be measured on a set of actual organisms. For example, to define a concept of fitness of a type in terms of the mean of a distribution of measurable token fitnesses is to define a concept of statistical type fitness. The same is true when fitness is defined in terms of variances, higher moments, or other statistical properties of a set of measurable token fitnesses. To give another example, when fitness is defined in terms of a regression of token fitnesses on the types of particular individuals, this is a statistical type fitness [see e.g., de Jong (1994) for comparisons of several such concepts]. Often researchers treat one or more statistical type fitness concepts as the only type fitness concepts characteristic of a population (e.g., Stearns, 1976). Note that the term “statistic” is sometimes restricted to functions applied to sample data from a larger population; I use the term “statistical type fitness” even when measurements on all members of a population are used to calculate a fitness measure.

It's worth mentioning cases in which what is under study is, in part, the response of a lineage to new mutations. For example, Lenski et al. (1991) compared growth rates of *Escherichia coli* lineages which differed only at a marker allele with no significant effect on fitness. The authors argued that differences in substitutions of new mutations arising in different lineages were responsible for differences in measured growth rates. One can view this experiment as aggregating fitnesses of different genotypes resulting from a number of mutations, but one purpose of the experiment was to record fitnesses for distinct lineages arising from the same initial type. What is being measured in this case, then, are statistical type fitnesses of the whole genome, including its capability for changes in epistatic interactions as new mutations arise.

### 2.4. Parametric type fitness

Sometimes researchers view a statistical type fitness as a way of estimating some underlying fitness value characteristic of a type. For example, Byars et al. (2010), mentioned above, use LRS measurements to estimate an underlying selection gradient with respect to variation in traits such as height and cholesterol level. The regression coefficient in terms of which such a selection gradient is described is a statistical type fitness, since it is defined by statistics on a set of measurable token fitnesses. However, Byars et al. treat this coefficient as an estimator of something else: this is an underlying selection gradient that varies with height, cholesterol level, etc., but which is not measured directly. To treat a fitness as an estimator of some kind is, implicitly at least, to have in mind an additional kind of fitness which plays a role like that of a statistical parameter of the situation. Fitness in the latter sense would capture an underlying fact about the population in its overall environment, defined partly in terms of probabilities (see below). In such cases, what is estimated is what I call a *parametric type fitness*. Note that the *estimator* is a statistical type fitness; the parametric type fitness is what is estimated. Typically, parametric type fitnesses are known only approximately via their estimators. The degree of certainty of our knowledge of them is given by the probabilities determined by the method of estimation and the data on which the estimate is based.

Remarks by Elena and Lenski (2003) also suggest the idea of a parametric type fitness. These authors discuss experiments such as (Lenski et al., 1991), mentioned above, in which the fitnesses of distinct populations of asexual microorganisms are compared. Elena and Lenski explicitly describe these fitnesses as properties of types (p. 458), and since these fitnesses are determined by measuring the growth rate of an entire population, they are statistical type fitnesses. However, Elena and Lenski remark that such a fitness measure “reflects the propensity to leave descendants” (Elena and Lenski, 2003, p. 458). The use of the word “propensity” suggests that the measured fitnesses are being viewed as estimating an underlying tendency of a type. (I don't assume that Elena and Lenski intend “propensity” in the sense described below.)

Researchers may not always make it clear whether a statistically defined fitness is being treated as a value which is itself of interest, or is being treated instead as an estimator of a value which is not directly observable. However, the distinction is sometimes explicit, as in (Byars et al., 2010), noted above, and is sometimes reflected mathematically. A particularly clear illustration of a mathematical distinction between measured and estimated quantities is provided by Weir and Cockerham's (1984) widely-used (Weir and Hill, 2002) methods for estimating F-statistics such as *F*_*ST*_, which can be used to provide evidence of natural selection under some circumstances. [For example, a common class of methods use *F*_*ST*_ values to show that the genetic differentiation between two populations at a particular locus is unusual relative to other loci. This provides evidence of selection if the extreme *F*_*ST*_ value can be shown to be improbable without past selection (e.g., Holsinger and Weir, 2009).] In Weir and Cockerham's approach, the way in which F-statistic values are calculated differs depending on whether they are intended to characterize a difference between the measurements of two or more populations, or instead are intended to characterize an underlying difference that is estimated by those measurements but not directly observed (Weir, 1996, ch. 5; Holsinger and Weir, 2009; cf. Nei and Kumar, 2000, section 12.3). This example is discussed further below.

### 2.5. Purely mathematical fitness

All of the preceding concepts should be distinguished from what I'll call *purely mathematical* fitness concepts. Many fitness concepts are used in mathematical models and computer simulations in ways which are neutral with respect to the distinctions described above. When developing or investigating implications of a mathematical or computer model, it's often unnecessary to specify whether fitnesses appearing in the model must be defined in terms of statistics on collections of individuals within populations, or should instead refer to some underlying parameter. Models are often implicitly used as if they abstract from such distinctions, treating fitness as a primarily mathematical concept. For example, a model may define the relative fitness *w*_*AA*_ of a genotype *AA* as any number between 0 and 1, inclusive, which then may be multiplied, in certain formulas, by the number of *AA* individuals (e.g., Nagylaki, 1992; Ewens, 2004; Rice, 2004). Some empirical applications of such a model will specify values for *w*_*AA*_ by calculating statistics derived from measurable token fitness values, but the mathematical model as such does not require that method of determining values for *w*_*AA*_. The same model might also be interpreted, in some cases, as describing parametric fitnesses. It needn't even be clear whether fitnesses are properties of types or tokens. For example, consider fitnesses in the Robertson-Price identity (Lynch and Walsh, 1998, p. 46), a simple version of the Price equation (Price, 1970) (cf. de Jong, 1994; Rice, 2004):

$$\Delta \bar{z}=\text{cov}(w,z)=\text{E}(w_{i}-\bar{w})(z_{i}-\bar{z}).$$

The equation gives the change in the average value of a trait *z* from one generation to the next. Here *z*_*i*_ could refer to a characteristic of the *i*th individual; *w*_*i*_ then refers to individual's *i*'s token fitness. Alternatively, *z*_*i*_ could refer, for example, to the *i*th allele present in a haploid, asexual population, or to some other type; in that case *w*_*i*_ is a type fitness. [de Jong, 1994 p. 4), inspired partly by Byerly and Michod (1991), similarly distinguishes mathematical fitness concepts from those which might be attributed to real organisms, but does not clearly distinguish between token, statistical, and parametric fitness concepts.]

## 3. Natural selection

This section discusses the role of fitness in natural selection, and argues that it's reasonable to view natural selection as a cause of evolution. This discussion provides background for section 4, which will apply some of the ideas discussed in this section to the fitness concepts defined in section 2. Section 4.5 gives additional reasons to think that natural selection is a cause of evolution.

Darwin gave this now well-known characterization of natural selection in the first edition of *On the Origin of Species*:

Owing to this struggle for life, any variation, however, slight and from whatever cause proceeding, if it be in any degree profitable to an individual of any species, in its infinitely complex relations to other organic beings and to external nature, will tend to the preservation of that individual, and will generally be inherited by its offspring. The offspring, also, will thus have a better chance of surviving, for, of the many individuals of any species which are periodically born, but a small number can survive. I have called this principle, by which each slight variation, if useful, is preserved, by the term of Natural Selection, …. (Darwin, 1964 [1859], p. 61)

Lewontin provided this useful summary of Darwin's idea:

As seen by present-day evolutionists, Darwin's scheme embodies three principles …:

1. Different individuals in a population have different morphologies, physiologies, and behaviors (phenotypic variation).
2. Different phenotypes have different rates of survival and reproduction in different environments (differential fitness).
3. There is a correlation between parents and offspring in the contribution of each to future generations (fitness is heritable).

These three principles embody the principle of evolution by natural selection. (Lewontin, 1970, p. 1)

Lewontin's summary does not capture all of the ways that “natural selection” and “fitness” are used in evolutionary biology, and some aspects of the summary have been criticized (Godfrey-Smith, 2009), but it is a useful point of reference. We can compress the idea further: natural selection requires heritable variation in fitness. The Modern Synthesis added the idea that the foundation of inheritance is genetic transmission, and the authors of the Synthesis, such as Fisher, Wright, and Dobzhansky, devised mathematical means for characterizing the roles of genes in natural selection. Recently, some authors have argued that non-genetic inheritance may have played a significant role in evolution as well (e.g., Jablonka and Lamb, 2006).

Lewontin's summary is non-committal about whether there are causal dimensions to natural selection, referring only to variation, rates, and correlations. By contrast, Darwin sometimes seemed to write as if natural selection was a cause of evolutionary change. The quotation from the *Origin* continues as follows, comparing natural and artificial selection:

I have called this principle, by which each slight variation, if useful, is preserved, by the term of Natural Selection, in order to mark its relation to man's *power* of selection. We have seen that man by selection can certainly *produce* great results, and can adapt organic beings to his own uses, through the accumulation of slight but useful variations, given to him by the hand of Nature. But Natural Selection, as we shall hereafter see, is a *power incessantly ready for action*, and is as immeasurably superior to man's feeble efforts, as the works of Nature are to those of Art. (, p. 61, emphasis added)

The emphasized words suggest that Darwin may have viewed natural selection as a cause of evolution. However, as I'll note below, some authors have argued that natural selection should not be viewed as a kind of cause.

Note that contemporary population biologists do sometimes treat natural selection implicitly, or even explicitly, as a general kind of cause of evolution. The common description of natural selection as a “force” (e.g., Gillespie, 2004)—contrasted with other forces such as mutation and drift—illustrates the pull of the intuition that “natural selection” refers to causes of a certain type (cf. Sober, 1984). Of course it's often possible to identify more fine-grained causes in particular cases. Some authors who argue that natural selection is not a cause take this last point as one motivation for their view (e.g., Endler, 1986; Matthen and Ariew, 2009). I'll touch on some of these authors' arguments below. However, the fact that there are more specific facts responsible for changes in a population does not by itself show that natural selection is not a particular type of cause. We can take natural selection to depend on, be constituted by, to supervene on, or to be realized by more specific causal factors in particular cases. We can view particular cases, in which there is selection in a population, as instances of a general kind of causal pattern—natural selection—which provides a general type of explanation of various biological phenomena. Similarly, when a falling rock hits another object and causes it to move, we needn't distinguish between various ways in which the atoms of the two objects interact with each other.

One point in favor of considering natural selection to be a cause is this: a central claim of evolutionary theory is that natural selection provides a significant part of the explanation of various patterns we observe in nature. There are ongoing debates among philosophers of science about what scientific explanation consists in. This is not the place for a detailed discussion of these debates, but most authors would probably agree that causes often explain their effects. My point here is only that it's extremely plausible that the reason that natural selection provides an explanation of so many phenomena is that it's a general kind of cause of those phenomena. I give further reasons to think that natural selection should be viewed as a cause of evolution in section 4.5. [Woodward (2011) provides an overview of debates about scientific explanation.]

It's reasonable to think that heritable differences in fitness are the properties which embody the causal aspect of natural selection. On the other hand, Sober (1984), Endler (1986), de Jong (1994), Matthen and Ariew (2009), and Walsh (2007), among others have argued that at least some fitness concepts imply that fitness differences cannot ever be considered causes of evolution. Like “natural selection,” “fitness” is used in different ways in different contexts, but there are good reasons to think that there are senses of both terms which make them causal. Understanding the sense in which fitness differences can be viewed as causes would clarify the role of natural selection in explanations of evolution. It is therefore worthwhile to investigate whether and how fitness can be understood as a causal property.

## 4. Fitness differences as causes of evolution?

The preceding section argued that it's reasonable to view natural selection as a cause of evolution. Section 3 also noted the central role that fitness differences play in the concept of natural selection, and suggested that heritable differences in fitness embody the causal aspect of natural selection. Using the distinctions between fitness concepts defined in section 2, we can now discuss which fitness concepts might play the role required by natural selection. We can also ask whether fitness concepts of each kind are able to capture causal facts about processes of natural selection. I'll propose answers to these questions, arguing that fitness concepts in some of the classes cannot themselves play the role of fitness in natural selection. Some of these fitness concepts nevertheless play very useful roles in research. In section 5, I'll suggest that their utility derives from their relationship to other kinds of fitness which are central to processes of natural selection.

### 4.1. Purely mathematical fitness

De Jong distinguishes between “a fitness concept that refers to the functioning of an organism (or genotype or trait), and a fitness concept in population biology summarizing numerical processes” (1994, p. 4), claiming that fitness in the second sense does not cause natural selection. For example, de Jong argues that certain fitness measures defined in population biology (e.g., the Malthusian parameter) are such that fitness cannot cause natural selection. If the point is that the Malthusian parameter is purely mathematical, then it is being treated as what I am calling a purely mathematical fitness concept. There is a sense in which it's correct that purely mathematical fitness concepts do not capture something causal. This is the same sense in which vector addition is not causal, even when it is used to describe the interaction of Newtonian forces. Though the mathematical concept is not in itself causal, it can nevertheless be used to give a precise characterization of something which is fundamentally causal. Thus purely mathematical fitness concepts *as such* are not causal, because mathematical terms without an interpretation or application are, of course, merely mathematical (cf. Millstein et al., 2009). This point is worth emphasizing for the sake of clarity even if it seems trivial. (I'll point out below that it's also possible that de Jong intended fitness concepts “summarizing numerical processes” as statistical type fitnesses.)

### 4.2. Measurable token fitness

Measurable token fitnesses, such as measured lifetime reproductive success in (Byars et al., 2010), provide measurements of effects which are the result of numerous causal factors. For example, an LRS value for a particular organism can result from the interaction of numerous heritable traits, as well as phenotypic properties due to minor developmental idiosyncrasies, and from possibly unique details of environmental circumstances encountered by an adult. Disease transmission and predation might depend, for example, on sudden fluctuations in the wind at particular times. In cases in which measurable token fitnesses are sensitive to such minor variations in environment circumstances, differences between measurable token fitnesses of parents and their offspring may sometimes be large, just due to chance. This kind of event would happen when, for example, the offspring of a fertile individual is predated soon after reaching reproductive age. The existence of such events does not imply that heritability is low, since heritability concerns patterns in the entire population. Nor does the existence of a large difference between the realized reproductive success of a parent and its child imply that there is not selection for traits realized by both parent and child. What such examples show, however, is that differences in measurable token fitness are not the kind of fitness differences which are central to natural selection. Natural selection requires heritable variation in fitness (section 3), but heritability doesn't properly apply to measured token fitnesses considered individually. The real connection between measurable token fitness and natural selection derives from the former's indirect relationship to parametric type fitness via statistical type fitness.

### 4.3. Tendential token fitness

As explained above, I use “tendential token fitness” for fitness concepts which treat fitness as something like a causal tendency of a particular individual to have a certain degree of survival, reproductive success, etc. The idea is vague, but seems to have an intuitive pull for many.

The *propensity interpretation of fitness* (PIF) is an attempt to give this idea further specificity. If successful, the PIF would make fitness differences causal. The most careful PIF proposals, including those by Brandon (1978, 1990), Mills and Beatty (1979), and Ramsey (2006) seem to treat fitness as an expectation (or a function of expectation and variance) for numbers of a particular individual's offspring, or close descendants, perhaps also including relatives' descendants via inclusive fitness calculations. The idea that is specific to the PIF is that the probabilities which define the relevant expectations, variances, etc., are *propensities*. These are theoretically postulated indeterministic dispositions, or fundamentally probabilistic causal tendencies (Popper, 1959; Hájek, 2010). The core idea of propensity can be introduced by an example: lead has a normal, *deterministic* disposition to melt when heated above 621°C at room temperature. Popper (1959) proposed that this idea be extended to allow *indeterministic* dispositions, assumed to satisfy axioms of probability. The notion of a propensity is controversial (Eagle, 2004), but is taken seriously by many philosophers of science and some scientists. The PIF's claim is that there are propensities for a particular individual to produce certain numbers of offspring, or to produce other evolutionarily relevant outcomes. The PIF claims that these propensities help define a concept of fitness (Beatty and Finsen, 1989; Brandon, 1990; Ramsey, 2006). By definition, propensities are causal in nature, so the PIF would make tendential token fitness a causal notion.

There have been attempts to define tendential token fitnesses in other ways, such as Bouchard and Rosenberg's (2004) definition of fitness in terms of the degree to which design problems are solved by a particular organism's traits. These other conceptions of tendential token fitness are vague in many respects, but they might also make fitness into a causal notion. It might seem, then, that by understanding natural selection as depending fundamentally on differences in tendential token fitnesses, its supposed causal character could be elucidated. It appears that this is not a role that tendential token fitness can play, however.

Sober (1984) argued that fitness, viewed as a function of all traits of a particular individual, can have no causal role in the individual's reproductive success. The reason is that, often, only some of an individual's phenotypic properties end up actually playing a role in its survival and reproduction. In general, traits need not be selected for because they play a role in *every* individual's life. Rather, a trait will be selected for when it confers a benefit often enough that the trait's average benefit exceeds its average cost, to a greater degree than competing traits. However, this situation is consistent with the trait making no difference, positive or negative, in the lives of particular individuals. Thus, for example, a heritable trait which influences the production of pheromones might play no role in the mating of a particular individual if the individual dies early because another heritable trait reduces parasite resistance. The outcome would have been the same if the individual had had a trait with a different effect on pheromones. Sober would say that in such cases the parasite-resistance trait was a cause of the individual's LRS, but the pheromone-influence trait was not. Thus what Sober calls “overall fitness,” which would reflect all of the organism's traits (or at least heritable ones), does not itself play a causal role in the organism's reproductive success. Byerly and Michod (1991) made a point closely related to Sober's, arguing against a concept of overall fitness (“adaptedness”) because “there is no underlying property which serves as a common physical basis for dispositions of organisms” (p. 6) to have particular tendencies toward reproductive success in different environments.

Abrams (2007) made a different but somewhat related point. He argued that the minor differences in environmental circumstances which affect measured properties such as lifetime reproductive success (section 4.2) can also have a strong influence on tendential token fitness. Abrams argued that most physiological processes and ecological interactions involve such a low degree of stochasticity, once details of surrounding circumstances are fixed, that each such interaction is effectively deterministic. According to this view, the variation in reproductive success that can be seen, for example, even for plant clones in superficially identical experimental treatments (e.g., Antonovics et al., 1987) is the result of minute, unobserved variation in distribution of substances in soil, differences in water flow, etc. All of these factors could be identified in principle, even though they may be difficult to identify in practice. Abrams argued, in effect, that variations between outcomes in such experiments would not be the result of a *fundamental* stochasticity. (By “fundamental stochasticity” I refer to variation that is not subject to further explanation, even in principle. For example, according to many physicists, variation in outcomes due to quantum mechanical effects has no further explanation.) However, if an individual's LRS, for example, is the nearly deterministic result of the specific circumstances in which it happens to find itself, then tendential token fitnesses will correspond nearly exactly to measurable token fitnesses: the only thing that can happen, given the circumstances, is what does happen, to a high degree of approximation. To the extent that this is so, the argument in section 4.2 that differences in measurable token fitness cannot play a central role in the concept of natural selection would apply to tendential token fitness as well. Ariew and Ernst (2009) give additional arguments against tendential token fitness.

### 4.4. Statistical type fitness

Many of the most useful fitness concepts are defined in terms of statistics on sets of measurable token fitnesses—i.e., on measurements of individual organisms in actual populations (e.g., Spitze, 1993; Cooper et al., 2003; Shaw et al., 2008; Byars et al., 2010). These are what I have been calling statistical type fitnesses, and are illustrated by numerous concepts defined by Stearns (1976, 1992) and de Jong (1994). Can fitness differences in this sense be considered causes of evolution? De Jong's remark (1994, p. 4), mentioned above, that certain fitness concepts “summarizing numerical processes” don't allow fitness to cause evolution may refer to statistical type fitness rather than to purely mathematical fitness.

Philosophers of biology who have come to be known informally as “statisticalists” argue that numerical concepts of fitness are not causal (e.g., Matthen, 2009; Matthen and Ariew, 2009; Walsh, 2007, 2010). I won't review these arguments—which go further than Endler's (1986) argument that natural selection is not a “force”—nor the large number of responses to them (e.g., Reisman and Forber, 2005; Haug, 2007; Shapiro and Sober, 2007; Millstein et al., 2009; Northcott, 2009; Otsuka et al., 2011; Abrams, 2012a). I note, however, that although statisticalists do not make all of the distinctions made here, some of their arguments do seem to focus on what I am calling statistical type, or possibly purely mathematical fitness concepts.

In my view, there are two fundamental reasons that natural selection, considered as a cause of evolution, cannot be understood as depending on statistical type fitnesses. First, statistical fitnesses are merely means for describing the way that a population is in fact changing over time. As such, they cannot also be taken as causing those very same changes.

Second, one can extend a point made above about measurable token fitness in section 4.3 to statistical type fitness. I pointed out that measurable token fitnesses of parents and offspring can sometimes be very different due to the details of interactions with environmental circumstances. Statistical type fitnesses are mathematical functions of measurable token fitnesses. As a result, statistical type fitnesses might occasionally differ greatly between generations (or nearby times) due to “coincidences” in which the measurable token fitnesses in one generation just happen to be very different from those in the next generation. Such events will be very improbable for heritable traits, especially for statistical type fitnesses calculated from a large number of properly sampled individuals. No special sort of instability in the environment is required in order for such a coincidence to occur, though. Coincidences in which statistical type fitnesses change radically can occur as long as we allow the possibility that the sorts of natural environments which are routinely considered to be stable are also complex. That complexity introduces the possibility that idiosyncratic, low-probability outcomes will occasionally occur. [Here I rely on normal intuitions about what counts as a stable environment; a more systematic discussion of stability isn't needed to make my point. Abrams (2009b,c) outlines a somewhat more precise concept of stable environment.]

Consider, for example, a diploid annual plant with wind-borne seed dispersal and a locus with two alleles *A* and *a* affecting root structure. It could occur, purely by chance, that in one year only seeds homozygous for *A* fall in areas suitable for germination, while in another year only *a* homozygotes do so. The statistical type fitnesses of *A* and *a* would thus change radically from one year to the next. Such improbable events presumably happen once in while in some populations, since they do have some small probability. [It's worth noting that less extreme “coincidences” may not be as rare as one might assume. Steiner and Tuljapurkar (2012) have argued that significant variation in measurable token fitnesses such as LRS, resulting from stochastic variation in environmental circumstances, is not uncommon.]

However, such rare, coincidental events, in which statistical type frequencies change radically, do not show that natural selection is not occurring with a consistent intensity and direction. Selection concerns the relationship between the environment and heritable types present in a population in that environment. That relationship does not preclude the possibility that the complexity of interactions between organisms and environmental circumstances gives rise to improbable coincidences. Of course, the improbability of such coincidences makes them almost completely irrelevant to empirical research. The point here is that statistical type fitnesses can fluctuate when selection pressure does not. This means that differences in statistical type fitnesses can occasionally differ greatly from the kind of fitness differences which are central to the concept of natural selection. Thus differences in statistical type fitnesses cannot play the central role needed by the concept of natural selection. Statistical type fitnesses are not part of what natural selection is.

### 4.5. Parametric type fitness

So far, I've argued that neither purely mathematical fitnesses, statistical type fitnesses, measurable token fitnesses, nor tendential token fitnesses are central to natural selection. I've also argued that fitnesses in the first three classes do not have causal properties. It remains to examine parametric type fitness concepts in detail. In this section I sketch a general conception of parametric type fitnesses that allows them to play the role of fitness in natural selection. This conception will also allow us to view natural selection as a kind of process that can cause evolution.

#### 4.5.1. Motivation

The fact that fitness values and other properties of selection processes are estimated by statistical methods, sometimes requires an assumption that there are probabilities concerning those natural processes whose properties are to be estimated. Consider, for example, the use of Weir and Cockerham's (1984) methods for estimating *F*_*ST*_ to provide evidence of natural selection (section 2). Weir and Cockerham's methods are based on the assumption that a natural population can be understood as having been sampled from a set of hypothetical populations, all of which are descended from a single population. More specifically, it's assumed that there is a probability distribution over these hypothetical populations, reflecting processes, including natural selection, that affect the sampling of alleles from ancestral populations (Weir, 1996, pp. 15ff., 46ff., 169ff.; Holsinger and Weir, 2009; Abrams, 2012a). The important point here is that this method of estimating *F*_*ST*_ requires the assumption that evolutionary processes, including natural selection, incorporate probabilities relevant to future representation in descendant populations. Without such an assumption, the mathematical form of the estimator would be different (Weir, 1996; Abrams, 2012a). Given the fruitfulness of such statistical methods, the assumption that there are probabilities involved in natural processes should be considered to be quite reasonable. I suggest that there are underlying parametric fitnesses definable in terms of such underlying probabilities, and that these fitnesses make it objectively more or less likely that frequencies of types within a population will follow certain trajectories over time.

Note that in many cases researchers do not explicitly treat statistical type fitnesses as estimates of an underlying parameter. I believe that a similar idea is sometimes present informally, however. For example, Pfennig (2000) measured differences in fertilization rate (as a component of fitness) between pure and hybrid matings, for two species of spadefoot toad. Pfennig did not estimate fitness values as statistical parameters of the underlying populations from these and other experimental measurements. However, based on the experiments she performed, Pfennig argued for general conclusions about differences in behavior and fitness values for these two species, under distinct conditions of allopatry and sympatry. One can interpret these as informal conclusions concerning parametric type fitnesses of some unspecified kind.

#### 4.5.2. Probability and causation

We might view a population and its environment at a given time as represented by a point in a high-dimensional state space. This state space would represent the genotypes, phenotypes, locations, and internal physiological states of members of the population and of other organisms in the environment. It could also include the positions and states of abiotic elements that might affect survival and reproduction of members of the population. The state space would thus include all combinations of conditions which are relevant to the evolution of the population over the period of time under study (cf. Abrams, 2009a,b). We can then think of changes in the population and environment as a trajectory through this state space. Such a state space would be too complex to model in any detail, of course, but I believe the idea of it is conceptually useful. (It is useful in the way that Hutchinson's (1957) concept of a niche was. Hutchinson's niche was a region in a high-dimensional space of ecological conditions, where conditions in that region allowed persistence of members of a given species. Though not useful for empirical applications, the idea helped organize thinking about practical theory and methods in ecology.)

For competing heritable types, the population at any one time will include a distribution of organisms with those types. Parametric fitnesses then summarize probabilities of possible trajectories through the state space: for example, trajectories in which higher-fitness types increase in frequency would be more probable. (Where there is frequency dependence, density dependence, or dependence of fitnesses on large-scale variation within the overall environment, such probabilities would also depend on how organisms with given types are distributed.) Though a natural population will only follow one trajectory, probabilities of trajectories can inferred by various means. For example, probabilities of trajectories can sometimes be inferred by conducting experiments with multiple populations in similar conditions.

My focus will be on computer simulations of a kind widely used in applications of population genetics. Such simulations allow investigating probabilities of population trajectories, or at least summary properties, such as fitnesses. For example, after fixing a simulation model's parameters—e.g., recombination rate, fitnesses, population sizes at times *t*_*i*_—a researcher will allow simulated populations to evolve repeatedly, often for several thousand runs with each set of parameters. Each combination of model parameters thus produces a distribution of trajectories of simplified, simulated populations. Each such trajectory represents ways that a population could evolve under the conditions represented by the specified parameters. These distributions of trajectories are then used to estimate probabilities of similar trajectories in natural populations. For example, such methods can be used to estimate the probability that measured SNP data is the result of natural selection rather than drift (e.g., Sabeti et al., 2007; Scheinfeldt et al., 2011; Huff et al., 2012).

However, the fact that these simulations are considered informative about real populations assumes, in effect, that if it were possible to go back and change past parameters for real populations, doing so would have altered probabilities of trajectories. That is, had past parameters been different, probabilities of past population trajectories would have been different as well. It is this assumption that allows us to draw inferences from simulations about whether observed data is or is not likely to be the result of past natural selection. For example, by manipulating simulated fitness parameters along with other parameters, it's possible to see whether known data is likely to have been produced by certain combinations of fitnesses under various conditions.

However, by treating hypothetical manipulations of fitness as ways of manipulating probabilities of population trajectories, we are treating fitnesses as having the role of causes (Woodward, 2003). That is, if fitnesses can control probabilities of various evolutionary outcomes, then they have the causal power to control the evolution of a population. Fitnesses then play a role analogous to the way in which initial conditions in a physics experiment sometimes control outcomes: the fact that we can manipulate physical outcomes by manipulating initial conditions provides evidence that in such cases, initial conditions are among the causes of the outcomes. Similarly, that we can manipulate probabilities of population trajectories—as evidenced by frequencies of population trajectories in simulations—shows that fitness differences play a role in causing trajectories (Abrams, 2012a). [This argument parallels, in some respects, arguments that experiments on replicate experimental populations under modified conditions can show that fitness differences are causes of evolution (Reisman and Forber, 2005; Shapiro and Sober, 2007).]

Given the discussion in this section and preceding ones, the following claims seem plausible:

- Natural selection involves differences in parametric type fitnesses.
- Parametric type fitnesses reflect objective probabilities concerning possible trajectories of a particular population in a particular kind of environment.
- These probabilities capture causal facts about a population and its environment.
- As a result, differences in parametric type fitnesses, and hence natural selection can be considered causes of evolution.

#### 4.5.3. Population and types

There is a puzzle suggested by the preceding claims. Parametric type fitnesses are properties of heritable types present in a population; they are not properties of individuals. Each individual typically is an instance of many such types, but as Sober (1984) argued (section 4.3) these types don't always make a difference in every individual's life. Moreover, I've argued that token fitnesses do not have a direct causal role in natural selection. Surely, though, it is only through individuals that types can affect evolution. How exactly does the fitness of a type play a causal role in evolution?

One answer that has been given is that what I call parametric type fitnesses are mathematical functions of tendential token fitnesses. For example, Mills and Beatty (1979) and Sober (1984) defined the fitness of a type as a mean of the tendential token fitnesses of the members of a population which realize that type at a given time. However, this sort of proposal may have a problem. Abrams (2007) argued that tendential token fitnesses would usually be very close to measurable token fitnesses (see section 4.3). If this is correct, then if parametric type fitnesses were defined in terms of the tendential token fitnesses of organisms actually present during a period of time, the former fitnesses would have a problem like that attributed to statistical type fitnesses in section 4.4: parametric type fitnesses would occasionally fluctuate in ways inappropriate to the role of fitness differences in natural selection (Abrams, 2007).

Abrams (2007) allowed that a modified version of the same strategy for defining parametric type fitness might work. Rather than defining a type fitness as a mathematical function of tendential token fitnesses of organisms actually present in a population during a given period of time, the parametric fitness of a type could be defined by a function of tendential token fitnesses for a broader class of organisms. These could be tendential token fitnesses of possible organisms which realize the type, in possible environmental circumstances consistent with the overall character of the environment. Such combinations of organisms and circumstances might be infinite in number, and would usually not be equally probable. Thus what would be needed to make sense of this way of defining parametric type fitnesses would be probabilities of such combinations. This brings us back to the idea that parametric type fitnesses depend on probabilities of trajectories of a population and environment through a complex state space. Such probabilities would imply probabilities of combinations of possible organisms in possible environmental circumstances, since each trajectory includes a set of organisms in various environmental circumstances, at every moment. However, it may not be necessary to think of parametric type fitness as defined in terms of measurable token fitnesses. Fitness differences could be viewed as differences in probabilities of trajectories with different numbers of individuals of competing heritable types. If a type *A* is fitter than another type *B*, for example, that might mean that trajectories in which *A*'s increase in frequency and *B*'s decrease in frequency have greater probability. No mention of token fitnesses need be made.

This picture is loosely analogous to the following: consider a group of rocks evenly distributed, all at the same height, along the side of a steep, rocky hillside. If simultaneously given a push, we can predict, with some confidence, that most of the rocks will end up at a significantly lower elevation than that for which the what the initial push accounts. This is not guaranteed, though; some rocks may stop very quickly. Whether they are likely to do so depends on their size, shape, and the topography of the hillside (cf. Sober, 1984, section 3.2). Here rock types are defined by their sizes and shapes, and the hillside's topography corresponds to the character of an environment. We can speak of the probability that rocks of given types will fall certain distances on a hillside with a given topography. We need not mention individual rocks in particular locations on a particular hillside, if we view a rock type as defining a set of probabilities of ways of falling down hills with a given kind of topography.

[Note that there are deep questions concerning the nature of the probabilities mentioned here (cf. Brandon, 1990; Millstein, 2003; Abrams, 2006, 2007; Hájek, 2010). My view is that these probabilities are what are called mechanistic or microconstant probabilities (Rosenthal, 2010; Strevens, 2011; Abrams, 2012b,c).]

## 5. Constraints and questions

Early in this paper, I remarked that an appropriate fitness measure depends on research questions and methodological constraints. The discussion above allows us to begin to clarify the senses in which fitness depends on these factors.

First, different type fitnesses may be relevant to different research questions. This point applies either to parametric type fitnesses or to the statistical type fitnesses used to estimate them. A simple illustration will convey the point: one type might be selected for under conditions which favor *r* selection, in which production of many offspring early in life is favored. Another type could be selected for later, after the carrying capacity *K* of the environment is reached. This means that questions about the evolution of a population during a short period before the carrying capacity is reached may require a different definition of fitness than questions about the evolution of the population over a longer period (Abrams, 2009b). We can view this as a situation in which the underlying causal factors are different for evolution over the different periods of time: over the longer period, resource limitations play a role in evolution of the population in a different way than they do in the early, shorter period. This is just one illustration, however. Further investigation of relationships between research questions and type fitnesses would be worthwhile.

Statistical type fitnesses are related to parametric type fitnesses in the following way: since in practice we can usually only estimate parametric type fitnesses, research requires statistical type fitness measures. But given that statistical type fitnesses are used to estimate the parametric type fitnesses, statistical type fitnesses depend on research questions in roughly the same sense that parametric type fitnesses do. (The preceding points are applicable whether the research questions concern changes in gene or genotype frequencies, or changes in phenotype distributions as in evolutionary quantitative genetics.)

However, statistical type fitnesses also depend on methodological constraints, as do measurable token fitnesses. These may include the amount and kind of data that it is possible to gather (e.g., how many measurements, at what times?), the cost of acquiring this data, the likelihood that it will be of such and such quality, etc. Methodological constraints also include the statistical and computational methods available, and their costs. We want our results to have a certain degree of validity, so there may be tradeoffs between choices of different statistical or empirical methods. All of these factors are relevant to how we define statistical type fitnesses and the measurable token fitnesses on which they depend.

Purely mathematical fitnesses provide part of the mathematical framework underlying some of estimation procedures. Mathematical models can also be used to investigate empirical populations (see above), but they can also be used to understand how populations would be likely to evolve, independent of our measurement of them.

In the preceding sections I suggested that probabilities on which parametric fitnesses depend are probabilities relative to a particular population (beginning from a particular time) in a particular environment. There is a sense in which both research questions and methodological constraints play a role in specifying what counts as a relevant population. First, though populations are not arbitrary collections of individuals, there is some leeway in how they are defined. This is illustrated, for example, by choices made by researchers using human genome data such as the Human Genome Diversity Panel (HGDP-CEPH) (Li et al., 2008). For example, Moreno-Estrada et al. (2009) grouped HGDP-CEPH data into seven populations for some analyses, and 39 for others, in part because of population size requirements for different statistical tests. This illustrates an interaction between research questions and methodological constraints, because one obviously can't answer questions about certain populations unless methods and data allow the possibility of answering them. On the other hand, though the choice of what counts as a population can reflect methodological constraints, I suggest that once the choice of time period and population or populations has been determined, methodological constraints play no role in determining the nature of relevant *parametric* type fitnesses. These fitnesses concern processes in the world relative to the specified population, and the environment with which it interacts in a given period of time; parametric type fitnesses do not depend on how we measure them.

Moreover, by choosing the individuals which make up the population studied, and also choosing to study a particular a period of time over which evolution takes place, one implicitly chooses a range of environmental variation (Abrams, 2009c). For example, a population evolving over 100 years might experience a different range of environment variation than the same population over a single year. Thus fitnesses affecting the probable changes in frequencies of the same set of types may be different depending on whether research questions concern one period of time or another.

However, I suggest that the fact that researchers' choices affect what sorts of conditions enter into parametric fitnesses does not take away from their objectivity: processes in the world determine a variety of objective fitness measures, each relative to populations, time periods, and environmental conditions. Research questions determine which of these objective fitnesses are of interest to us. Methodological constraints then determine the appropriate methods, involving particular statistical type fitnesses and other quantities, for estimating the relevant parametric type fitnesses.

## 6. Conclusion

I've given a new classification of fitness concepts, and argued that this classification can be used to clarify different roles that these concepts play in theory and research. Statistical type fitness is defined in terms of measurable token fitnesses, and is used to estimate parametric type fitness. Tendential token fitness might function as a component of parametric type fitness, or might be a theoretical notion whose connection to natural selection is not clear. Purely mathematical fitnesses are the mathematical shadows of other notions of notions of fitness, but are useful because of their role in defining and constraining fitness measures of other kinds.

I've argued that parametric type fitnesses are plausibly causal, and that it is parametric type fitness differences which provide the causal aspect of the process of natural selection in real populations. Statistical type fitnesses, measurable token fitnesses, and purely mathematical fitnesses have no causal properties as such. However, they are extremely useful for characterizing processes of natural selection, by indirectly characterizing parametric type fitnesses. In fact, it is usually only through the use of statistical type fitnesses in estimation that we have any access to parametric type fitnesses. The detailed character of parametric type fitnesses will often be unknown, but it is these fitnesses which, in effect, operate on real populations. In these respects parametric type fitnesses are no different from causal factors—for example, physical forces—in most real complex systems.

Finally, I've spelled out relationships between these different classes of fitness concepts, research questions, and methodological constraints. Among other things, I argued that while both research questions and methodological constraints can affect the choice of fitness measures other than parametric type fitnesses, relevant parametric type fitnesses depend only on research questions, once the population to be studied and its environment is specified.

I don't claim to have resolved all important issues concerning existing fitness concepts. I do believe, though, that this essay represents a step forward in our understanding of fitness concepts, their interrelationships, and their roles in research.

### Conflict of interest statement

The author declares that the research was conducted in the absence of any commercial or financial relationships that could be construed as a potential conflict of interest.
